# Stress Echocardiography in the Follow-Up of Young Patients with Repaired Aortic Coarctation

**DOI:** 10.3390/jcm13185587

**Published:** 2024-09-20

**Authors:** Giovanni Di Salvo, Jennifer Fumanelli, Serena Graziano, Alice Pozza, Irene Cattapan, Sara Moscatelli, Biagio Castaldi, Domenico Galzerano

**Affiliations:** 1 Pediatric Cardiology and Adult Congenital Unit, Department of Women’s and Child’s Health, University of Padua, 35121 Padova, Italy; jennifer.fumanelli@aopd.veneto.it (J.F.); serena.graziano@studenti.unipd.it (S.G.); irene.cattapan@aopd.veneto.it (I.C.); b.castaldi@yahoo.it (B.C.); 2 Working Group on Congenital Heart Disease and Cardiovascular Prevention of the Italian Society of Cardiology, 00136 Roma, Italy; dgalzerano@kfshrc.edu.sa; 3 Heart Center, King Faisal Specialist Hospital and Research Center, Riyadh 12611, Saudi Arabia

**Keywords:** coarctation, stress echocardiography, hypertension

## Abstract

**Background:** Aortic coarctation (CoA) is a congenital heart disease affecting 5–8% of patients, with long-term complications persisting despite successful correction. Stress echocardiography (SE) is increasingly used for evaluating cardiac function under stress, yet its role in repaired CoA remains under-explored. **Objective:** This study aimed to assess the predictive value of SE and myocardial strain in repaired CoA patients with a history of hypertension without significant gradients or with borderline gradients at rest. **Methods:** Between June 2020 and March 2024, we enrolled 35 consecutive CoA patients with successful repairs and either a history of hypertension or borderline Doppler gradients. Baseline and peak exercise echocardiographic measurements, including left ventricular mass index (LVMi) and global longitudinal strain (LVGLS), were recorded. Patients were followed for up to 4 years. **Results:** At baseline, the positive SE group had higher systolic blood pressure (SBP) and diastolic blood pressure (DBP) compared to the negative SE group. The positive SE group also exhibited significantly higher basal and peak trans-isthmic gradients. Positive SE was found in 45.7% of patients, with 68.7% of these requiring re-intervention during follow-up. A peak trans-isthmic gradient > 61 mmHg during exercise predicted recoarctation with 100% sensitivity and 71% specificity (AUC = 0.836, *p* < 0.004). **Conclusions:** SE identifies at-risk patients post-CoA repair, aiding in early intervention. A peak trans-isthmic gradient > 61 mmHg during exercise is a strong predictor of recoarctation. These findings support incorporating SE into routine follow-up protocols for CoA patients, particularly those with a history of hypertension and borderline gradients, to improve long-term outcomes and quality of life.

## 1. Introduction

Aortic coarctation (CoA) is a well-known congenital heart disease (CHD), representing approximately 5–8% of all congenital heart diseases, with a higher prevalence in males [1]. Despite successful correction, this population experiences reduced life expectancy due to mid- and long-term complications [1]. CoA is also considered a general arteriopathy due to modifications to aortic wall elastic properties since the fetal period [2]. Even with early repair, hypertension remains a frequent complication [3], and aortic elastic abnormalities persist into adulthood despite neonatal correction [4]. Recoarctation is one of the most feared complications, often necessitating re-intervention to reduce left ventricular (LV) pressure overload [5]. Abnormal myocardial deformation indices, such as strain and strain rate, are reduced even in patients with successful CoA repair [6]. Recoarctation is defined as: hypertension with a clinical gradient between upper and lower limbs > 20 mmHg confirmed with invasive measurement (peak to peak > 20 mmHg) [6]. However, in clinical practice, CoA patients who are hypertensive but without a significant gradient, or with a borderline gradient without hypertension, are frequent [7]. The decision-making in this borderline situation is unclear and the role of non-invasive tests is debated [7].

Stress echocardiography (SE) is a widely used tool to assess various cardiac diseases in the adult population [8,9,10]. Recently, it has gained consensus as part of the diagnostic–prognostic work-up in adult congenital heart diseases [10]. SE allows for simultaneous assessment of myocardial function and hemodynamics under physiological or pharmacological conditions, including in CoA patients. However, data on cardiac function and on trans-isthmic gradient during exercise in repaired CoA patients remain limited.

Thus, the aim of our study is to assess the predictive value of SE and myocardial strain in repaired CoA patients with a history of hypertension without a significant gradient or with a borderline gradient at rest.

## 2. Methods

### 2.1. Study Population

Between June 2020 and March 2024, we prospectively enrolled 35 consecutive CoA patients, regularly followed at our pediatric cardiology and adult congenital heart disease tertiary center. Inclusion criteria included isolated coarctation and successful repair (surgical or interventional), defined as a post-procedure invasive gradient ≤ 20 mmHg, with no significant valvular heart disease (only mild cases were included), either a history of hypertension in the presence of a trans-isthmic Doppler mean gradient ≤ 20 mmHg, or without hypertension but with a borderline Doppler gradient at rest > 20 mmHg and ≤40 mmHg, without diastolic drugs and without clinical gradient. Exclusion criteria included arrhythmias, paced rhythm, valvulopathy more than mild, and a height below 130 cm (required to use the semi-supine bicycle).

Anthropometrical and clinical data were collected at the time of stress echocardiography evaluation, including age, gender, body surface area (BSA), systolic and diastolic arterial pressure at rest and during exercise, history of systolic hypertension, type and timing of correction (surgical vs. endovascular), and symptoms at rest and during physical activity. The definition of systolic hypertension at rest and at peak exercise in children and adults is based on the respective current guidelines.

The study was performed in line with the principles of the Declaration of Helsinki and was approved by the institutional ethics committee. As this study involved the retrospective analysis of data collected during clinical activity, the institutional review board waived the need for patients to provide written informed consent.

### 2.2. Baseline Echocardiography and Stress Echocardiography Study

All patients underwent a basal transthoracic echocardiography using a GE Vivid E95 machine (GE Healthcare, Wauwatosa, WI, USA) with an M5S ultrasound transducer. Standard parasternal short- and long-axis, apical 2-3-4-dimensional chambers, suprasternal view of the arch, and color-guided pulsed- and continuous-wave Doppler images were acquired at rest in the left lateral decubitus or supine position. Blood pressure at rest was measured at pre- and post-ductal sites before performing the basal echocardiography.

Stored images were analyzed and post-processed using EchopacTM software (EchoPac version 204, GE Healthcare). Measurements included LV wall thicknesses, LV mass index, LV systolic function through Simpson’s biplane method, LV diastolic function (E/A and E/E′ index), aortic valve gradient and/or regurgitation, and peak and mean gradient across the descending aorta, following European Association of Echocardiography and European Association of Cardiovascular Imaging (EACVI) guidelines. An expert operator performed speckle tracking echocardiography (STE) on stored DICOM echo clips using the EchoPac software (EchoPac version 206, GE Healthcare). LV GLS was analyzed on standard gray-scale images in the apical 2-chamber, 3-chamber, and 4-chamber views with a frame rate of 50–80/s. Echocardiographic measurements were repeated at peak effort.

Patients underwent an exercise test according to the Bruce protocol to study cardiac performance during maximal exercise. This protocol involves using a semi-supine bicycle with increasing cycling power of 25 watts every 2 min. During the exam, a 12-lead ECG and blood pressure were continuously monitored non-invasively. Systolic–diastolic pressure values were detected every two minutes by a sphygmomanometer connected to a cuff positioned at the right arm (pre-ductal site), and heart rates were recorded simultaneously.

We defined a positive stress echocardiographic exam [8] as an average trans-isthmic gradient under stress ≥ 30 mmHg (major criterion), associated with at least one of the minor criteria: hypertensive response to effort, appearance of diastolic run-off at the descending aorta, and/or abdominal level.

### 2.3. Follow-Up

Patients were followed for up to 4 years (range 6–48 months). Recoarctation was defined according to ESC guidelines [6] as: hypertension with a clinical gradient between upper and lower limbs > 20 mmHg confirmed with invasive measurement (peak to peak > 20 mmHg).

### 2.4. Statistical Analysis

Statistical analysis was performed using MedCalc^®^ Statistical Software version 22.023 (MedCalc Software Ltd., Ostend, Belgium; https://www.medcalc.org; 2024).

Categorical variables were reported as percentages (%), while continuous variables were presented as mean ± standard deviation. The Shapiro–Wilk test and histogram were used to verify normality for each variable. Student′s t-test was performed for normally distributed continuous variables, while the Mann–Whitney U test was used for nonparametric continuous variables. The chi-square test was used for categorical variables to test for significant differences between groups. ROC analysis was performed to identify the best cut-off value. Statistical significance was attributed to *p*-values < 0.05.

## 3. Results

### 3.1. Baseline Characteristics

The study population consisted of 35 children and young adults with an average age of 23.8 ± 12.8 years, of which 24 (68.6%) were male (Table 1). The average body surface area (BSA) was 1.69 ± 0.24 m^2^. Baseline systolic blood pressure (SBP) was 127.63 ± 14.99 mmHg, and baseline diastolic blood pressure (DBP) was 72.17 ± 8.8 mmHg. Common associated defects included bicuspid aortic valve in 24 (68.6%) patients, ventricular septal defect (VSD) in 12 (34.3%) patients, and various mitral valve anomalies. Mitral dysplasia was observed in three (8.6%) patients. The average age at correction was 2.36 ± 4.19 years. The primary types of correction were end-to-end anastomosis in 23 (65.7%) patients, percutaneous dilation with stent in 6 (17.1%), and percutaneous aortoisthmoplasty in 3 (8.6%).

A history of systolic hypertension was present in 20 (57%) patients, with anti-hypertensive therapy including beta-blockers (n = 4), ACE inhibitors (n = 2), ARI (n = 5), and calcium channel blockers (n = 1), or life-style recommendations (n = 9). None of the studied patients had a clinical gradient between right arm and right leg > 20 mmHg.

### 3.2. Baseline Echocardiographic Measurements

The baseline echocardiographic characteristics of the studied sample are presented in Table 2.

The left ventricular mass index (LVMI), as per the ASE guidelines, was increased (88.54 ± 26.92 g/m^2^). Twenty patients (55%) showed left ventricular hypertrophy. All patients presented normal systolic function at rest. Diastolic function at rest was within the normal range (8.1 ± 4.9). The baseline mean trans-isthmic gradient was 17.9 ± 9.2 mmHg, no patient had a olodiastolic drag at rest.

### 3.3. Echocardiographic Findings at Peak Exercise (Table 2)

Twenty-two patients reached at least 80% of the target heart rate (220-age), while in the remaining 13 patients the exam was stopped because of leg pain. None of the studied patients had symptoms like angina, dyspnea, or palpitations. ECG did not show any significant modification in the ST-T segment. No arrythmias, other than sporadic premature atrial beats, occurred during the exam.

In the studied sample, blood pressure and trans-isthmic gradient significantly increased at peak exercise (64.9 ± 27.2 mmHg, *p* < 0.0001). At peak exercise, diastolic run-off in the descending aorta became apparent in 48.6% of the population, while no run-off was seen in the abdominal aorta. Global systolic function, as assessed by LVEF, significantly increased at peak exercise. No changes were observed in LV GLS.

According to the guidelines, in our studied sample, 16 (45.7%) patients had a positive stress echocardiographic (PSE) evaluation [8]. Comparing baseline clinical and echocardiographic characteristics (Table 3), PSE patients showed higher basal SBP, were more frequently under hypertensive therapy (50% vs. 21%, *p* = 0.03). Left ventricular mass index (LVMi) was similar between groups (88.1 ± 28.8 g/m^2^ vs. 88.9 ± 24.4 g/m^2^, *p* = 0.93). The basal trans-isthmic gradient was significantly higher in the PSE group (22.6 ± 8.6 mmHg) compared to the NSE group (12.6 ± 6.2 mmHg, *p* = 0.002).

Peak SBP and DBP tended to be higher in the PSE group compared to the NSE group, although this difference was not statistically significant (*p* = 0.05).

Similarly, the peak trans-isthmic gradient was significantly higher in the PSE group (45.1 ± 12.1 mmHg) compared to the NSE group (22.4 ± 7.3 mmHg, *p* < 0.0001).

There were no significant differences in basal left ventricular ejection fraction (LVEF) between the PSE (63.2 ± 6.4%) and NSE groups (61.6 ± 4.2%, *p* = 0.37), nor in peak LVEF (66.1 ± 5.4% for PSE vs. 64.1 ± 4.4% for NSE, *p* = 0.279). Basal left ventricular global longitudinal strain (LVGLS) was also similar between the PSE (−17.6 ± 1.9%) and NSE groups (−18.1 ± 2.1%, *p* = 0.63), as was peak LVGLS (−18.7 ± 2.3% for PSE vs. −18.9 ± 1.9% for NSE, *p* = 0.75). Diastolic function (E\E′average) was not included in the analysis because at peak exercise the pattern was frequently fused.

**Figure 1:**
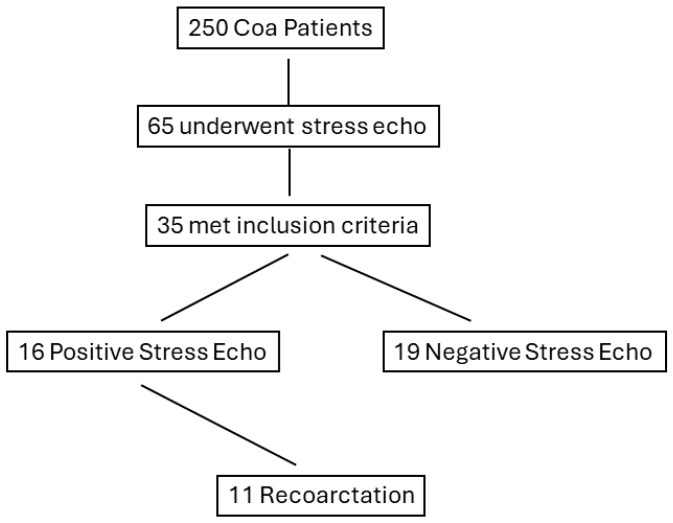
Flow chart displaying the stratification of patients based on positive response to stress echocardiography. CoA, coarctation of the aorta.

Among the 35 studied patients, there were 11 cases (31.4%) of recoarctation during follow-up, (mean duration (18 ± 14 months)) requiring percutaneous stent implantation (Figure 1). Two cases refused treatment and were left on anti-hypertensive medications and close follow-up. All 11 cases belonged to the PSE group (68.7%).

**Figure 2:**
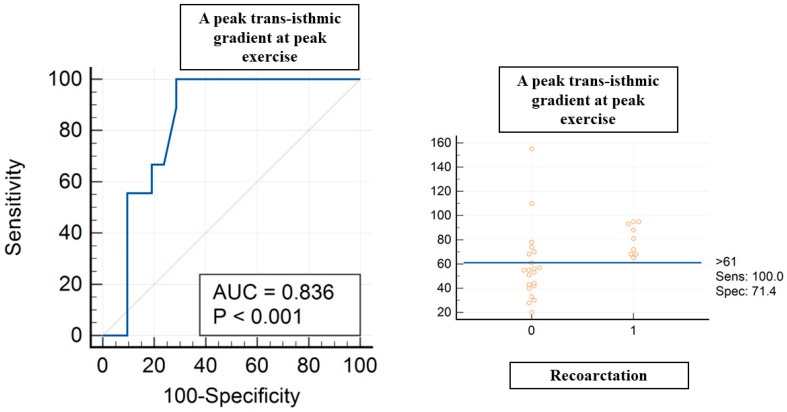
ROC curve analysis for peak trans-isthmic gradient at peak exercise and recoarctation. Red circles are coa patients, 0 without recoarctation, 1 with recoarctation.

A peak trans-isthmic gradient at peak exercise > 61 mmHg showed a sensitivity of 100% and specificity of 71% (AUC of 0.836, *p* < 0.004) in terms of predicting recoarctation (Figure 2). A mean trans-isthmic gradient > 34 mmHg showed a sensitivity of 77.8% and specificity of 71% (AUC of 0.765, *p* < 0.006) in terms of predicting recoarctation.

## 4. Discussion

Our study demonstrates that approximately 45% of children or young adults with apparently successfully repaired CoA have a positive stress echocardiographic evaluation. Notably, 68.7% of the patients with a positive stress echo required percutaneous intervention. Stress echocardiography plays a crucial role in the long-term monitoring of patients with various cardiac diseases [6]. However, its role in the follow-up of patients who have undergone successful repair of CoA remains unclear [7]. CoA patients, despite initial successful intervention, are at risk for complications such as recoarctation, hypertension, and heart failure, affecting long-term survival [1,2,3,4,5]. Thus, a better non-invasive test to identify at-risk groups could be beneficial.

The gradient observed during exercise is predictive of recoarctation [6]. This finding is critical as it provides a quantifiable measure that can be monitored over time, allowing for timely identification and management of recoarctation, preventing adverse outcomes and preserving long-term cardiovascular health in these young patients. At rest, an isthmus gradient may be absent due to collaterals, but during exercise, the increased cardiac output may overwhelm the collaterals, revealing the isthmus gradient.

Our findings of a mean trans-isthmic gradient at peak exercise > 34 mmHg predictive of recoarctation are in keeping with guidelines that suggest a cut-off of 30 mmHg [6]. However, in our study, a peak trans-isthmic gradient at peak exercise > 61 mmHg showed a better accuracy. However, measurements of peak velocity, especially at peak exercise, can be challenging and more subjective.

Patients with a history of hypertension and higher baseline isthmus gradient were more frequently positive at stress echo. Previous studies using pharmacological or physical stress echo have demonstrated a positive correlation between systolic blood pressure and trans-isthmic gradient in CoA patients [10,11].

A recent study demonstrated that hypertensive response to exercise in CoA patients is not predictive of cardiovascular events [12]. Interestingly, in our study, a positive stress echo was found in all patients who developed during follow-up an indication for percutaneous treatment. These findings suggest that it is not the hypertensive response per se, but the combination between peak exercise trans-isthmic gradient and hypertensive response, that may hold a prognostic value.

At peak exercise, LV GLS was not different between PSE and NSE patients. This finding is somehow surprising since several papers demonstrated LV GLS to be able to early detect subclinical dysfunction [5]. This is likely due to technical limitations in measuring LV GLS at peak exercise. The speckle-tracking software processes video at a frame rate between 50 and 100 bpm, which is suitable for resting heart rates but inadequate for peak exercise heart rates, leading to under-sampling and less accurate measurements [13]. However, LV GLS in both groups did not show a physiological increase at peak exercise in both groups. This finding may suggest an early subclinical endocardial damage affecting longitudinally oriented fibers. The value of LV GLS, even in the presence of normal or recovered LVEF, in terms of predicting the deterioration of LV EF over time or cardiovascular events, has been consistently observed in several studies on congenital heart disease, regardless of whether patients had right or left heart lesions, suggesting LV GLS is a robust imaging tool for risk stratification [14]. The use of advanced echocardiography could be beneficial to also predict outcomes in CoA patients; however, some technical limitations for its application at higher heart rates need to be implemented [15,16].

**Limitations of the study.** This study carries several limitations. First, the sample size may seem small; however, it is in the range of previous studies on the same topic [7,17,18] and it reflects the selection criteria. Some of the results (LV GLS at peak exercise) may be influenced by technical challenges, but this reflects the limits of the current technology.

In conclusion, stress echocardiography in our experience has an important role in the follow-up of young patients with repaired CoA. It helps identify patients at higher risk for recurrence. This proactive approach may ensure timely intervention and improve long-term outcomes and quality of life for these patients. Our findings support the inclusion of stress echo in routine follow-up protocols for patients with repaired CoA, especially those with a history of hypertension and borderline baseline gradients.

## Figures and Tables

**Table 1:** General Characteristics of the Studied Sample.

Patients = 35	
**Age at Evaluation (years)**	23.8 ± 12.8
**Sex (male)**	24 (68.6%)
**Height (cm)**	169.3 ± 11.6
**Weight (kg)**	61.8 ± 15
**BSA (m^2^)**	1.69 ± 0.24
**BMI (kg/m^2^)**	21.5 ± 4.3
**SBP Basal (mmHg)**	127.6 ± 14.9
**DBP Basal (mmHg)**	72.2 ± 8.8
**Associated Defects**	Bicuspid Aortic Valve 24 (68.6%)
	Aortic Subvalvar Membrane 3 (8.6%)
	VSD 12 (34.3%)
	Mitral Valve Anomalies
	Parachute Mitral Valve 2 (5.7%)
	Mitral Arcade 1 (3.5%)
	Mitral Dysplasia 3 (8.6%)
	Isolated Cleft 1 (3.5%)
	Tricuspid Dysplasia 1
**Age at Correction (Years)**	2.4 ± 4.2

**Table 2:** The Baseline and Peak Exercise Echocardiographic Characteristics of the Studied Sample.

	Basal (n = 35)	Peak-Exercise (n = 35)	*p* Value
**SBP (mmHg)**	127.6 ± 15	168.5 ± 31.5	**<0.0001**
**DBP (mmHg)**	72.2 ± 8.8	87.4 ± 19.4	**<0.0001**
**HR (bpm)**	73.8 ± 17.7	141.0 ± 21.6	**<0.0001**
**Trans-Isthmic Gradient Mean (mmHg)**	17.9 ± 9.2	34.5 ± 15.5	**<0.0001**
**Trans-Isthmic Gradient Peak (mmHg)**	30.6 ± 9.9	64.9 ± 27.2	**<0.0001**
**Olo-Diastolic Run-Off at Descending Aorta**	0	17 (48.6%)	**0.0001**
**LVEDD (mm)**	46.6 ± 5.2		
**IVSDD (mm)**	9.8 ± 2.9		
**PWDD (mm)**	8.6 ± 1.6		
**LVMi (g\m^2^)**	88.5 ± 26.5		
**E\E′average**	8.1 ± 4.9	NA	
**LVEF (%)**	62.5 ± 5.7	65 ± 5.1	0.05
**LVGLS (−%)**	−18.5 ± 1.7	−18.8 ± 1.9	0.30

Legend: SBP: Systolic Blood Pressure; DBP: Diastolic Blood Pressure; HR: Heart Rate; LVEDD: Left Ventricular End Diastolic Diameter; IVSD: Interventricular Septum Diastolic Diameter; Posterior Wall Diastolic Diameter; PWDD: Posterior Wall Diastolic Diameter; LVMi: Left Ventricular Mass Index; LVEF: Left Ventricular Ejection Fraction; LVGLS: Left Ventricular Global Longitudinal Strain.

**Table 3:** Comparison of Clinical and Echocardiographic Data Between Patients with a Positive Stress Echo (PSE) and Patients with a Negative Stress Echo (NSE).

	PSE (*n* = 16)	NSE (*n* = 19)	*p* Value
**Age at Study (Years)**	25.5 ± 14.1	22.4 ± 11.1	0.489
**Age at Correction (Years)**	2.26 ± 3.0	2.44 ± 4.9	0.905
**Basal SBP mmHg**	133.6 ± 12.7	122.6 ± 14.6	**0.03**
**Basal DBP mmHg**	72.9 ± 6.4	76.6 ± 19.1	**0.03**
**Basal HR bpm**	70.4 ± 14.3	76.6 ± 19.1	0.34
**Hypertensive Therapy (%)**	8 (50%)	4 (21%)	**0.03**
**Peak SBP mmHg**	178.9 ± 25.9	159.8 ± 32.3	0.07
**Peak DBP mmHg**	94.1 ± 18.2	81.2 ± 17.9	0.05
**Peak HR bpm**	142.3 ± 20.5	139.9 ± 21.8	0.77
**LVMi g\m^2^**	88.1 ± 28.8	88.9 ± 24.4	0.93
**Rest-Trans-Isthmic Gradient (Peak) mmHg**	43.3 ± 19.1	24.6 ± 8.5	**0.003**
**Rest-Trans-Isthmic Gradient (Mean) mmHg**	22.6 ± 8.6	12.6 ± 6.2	**0.002**
**Peak-Exercise Trans-Isthmic Gradient (peak) mmHg**	81.7 ± 24.9	45.8 ± 13.9	**<0.0001**
**Peak-Exercise Trans-Isthmic Gradient (mean) mmHg**	45.1 ± 12.1	22.4 ± 7.3	**<0.0001**
**Basal LVEF (%)**	63.2 ± 6.4	61.6 ± 4.2	0.37
**Peak LVEF (%)**	66.1 ± 5.4	64.1 ± 4.4	0.279
**Basal LVGLS (−%)**	−17.6 ± 1.9	−18.1 ± 2.1	0.63
**Peak LVGLS (−%)**	−18.7 ± 2.3	−18.9 ± 1.9	0.75

Legends: SBP: Systolic Blood Pressure; DBP: Diastolic Blood Pressure; HR: Heart Rate; LVMi: Left Ventricular Mass Index; LVEF: Left Ventricular Ejection Fraction; LVGLS: Left Ventricular Global Longitudinal Strain.

## Data Availability

Data will be available upon motivated request to the Authors.
